# Effect of SUV39H1 Histone Methyltransferase Knockout on Expression of Differentiation-Associated Genes in HaCaT Keratinocytes

**DOI:** 10.3390/cells9122628

**Published:** 2020-12-07

**Authors:** Barbara Sobiak, Wiesława Leśniak

**Affiliations:** Nencki Institute of Experimental Biology, Polish Academy of Sciences, 3 Pasteur Street, 02-093 Warsaw, Poland; b.sobiak@gmail.com

**Keywords:** CRISPR/Cas9, epidermal differentiation, histone modifications, H3K9me3, LCE1 genes, SUV39H1 histone methyltransferase

## Abstract

Keratinocytes undergo a complex differentiation process, coupled with extensive changes in gene expression through which they acquire distinctive features indispensable for cells that form the external body barrier—epidermis. Disturbed epidermal differentiation gives rise to multiple skin diseases. The involvement of epigenetic factors, such as DNA methylation or histone modifications, in the regulation of epidermal gene expression and differentiation has not been fully recognized yet. In this work we performed a CRISPR/Cas9-mediated knockout of *SUV39H1*, a gene-encoding H3K9 histone methyltransferase, in HaCaT cells that originate from spontaneously immortalized human keratinocytes and examined changes in the expression of selected differentiation-specific genes located in the epidermal differentiation complex (EDC) and other genomic locations by RT-qPCR. The studied genes revealed a diverse differentiation state-dependent or -independent response to a lower level of H3K9 methylation. We also show, by means of chromatin immunoprecipitation, that the expression of genes in the LCE1 subcluster of EDC was regulated by the extent of trimethylation of lysine 9 in histone H3 bound to their promoters. Changes in gene expression were accompanied by changes in HaCaT cell morphology and adhesion.

## 1. Introduction

Epidermal differentiation is a process through which keratinocyte progenitor cells, located at the basal layer contacting the dermis, acquire morphological and biochemical features necessary to form a tight and resilient barrier, which protects the organism against water loss and environmental hazards [1]. After an initial round of cell divisions, keratinocytes start to form tight intracellular connections through desmosomes and to synthesize components of the keratin cytoskeleton and the cornified envelope, which provide mechanical strength and impermeability. Eventually the cells flatten, become enucleated, and successively desquamate in the uppermost cornified layer. All these changes can be achieved thanks to the coordinated expression of multiple genes encoding essential structural proteins that often serve as markers of the ongoing differentiation process. Some keratinocyte-specific genes are grouped in clusters such as those comprising keratin-encoding genes on chromosome 12 [2] or the epidermal differentiation complex (EDC) on chromosome 1 in humans [3,4]. The latter gene cluster encodes 4 protein families: S100 proteins [5], S100 fused-type proteins (SFTP) [6], small proline-rich region (SPRR) proteins [7], late cornified envelope (LCE) proteins [8], and several other proteins such as loricrin (LOR) or involucrin (INV). Except for the S100 proteins, most others are components of the cornified envelope. Mutations in several EDC genes show strong association with various skin diseases [9].

Transcriptional regulation of epidermal differentiation has been extensively studied and several signaling pathways and transcription factors, among them p63 [10] and factors from the GRHL [11] and AP1 [12] families, were shown to be essential. Epigenetic regulation through DNA methylation, histone modifications, and miRNA activity may also be involved in this process [13]. Initial studies on DNA methylation of gene promoters revealed clear differences between undifferentiated and differentiated keratinocytes [14], although many gene promoters and putative regulatory regions within EDC were shown to maintain their methylation status during differentiation [15,16]. Lack of major changes in DNA methylation within EDC and elsewhere in the genome was recently confirmed by a genome-wide methylation analysis of in vitro differentiating keratinocytes [17]. ChIP-seq analysis of histone modifications revealed no major differences in chromatin configuration between progenitor epidermal cells and differentiated keratinocytes at promoter regions despite pronounced changes in gene expression [18]. However, a knockout of Ezh2 histone methyltransferase, which led to a reduced level of inhibitory histone mark H3K27me3, resulted in increased expression of some differentiation markers in mouse epidermis, including several EDC genes such as the one encoding loricrin or genes from the LCE1 subcluster [19].

In this work, we studied the role of H3K9 trimethylation (H3K9me3), an inhibitory histone modification, in the expression of EDC and other genes associated with keratinocyte differentiation. To do so, we performed a CRISPR/Cas9-mediated knockout of *SUV39H1*, encoding a H3K9 histone methyltransferase in HaCaT cells that originated from spontaneously immortalized human keratinocytes and preserved essential features of the founding cells, including the ability to differentiate [20]. We established that *SUV39H1* knockout caused a marked decrease in the level of H3K9me3 and resulted in altered expression of some but not all of the examined differentiation-associated genes. Using chromatin immunoprecipitation (ChIP), we examined whether increased expression of LCE1 genes correlated with changes in the level of H3K9me3 and other modified histones bound to their promoters. We also studied the impact of *SUV39H1* knockout on HaCaT cell adhesion, including cell-to-cell adhesion.

## 2. Materials and Methods

### 2.1. Cell Culture

HaCaT cells, spontaneously immortalized human keratinocytes (Cell Line Service, Eppelheim, Germany), were cultured in DMEM (Sigma Aldrich, St. Louis, MO, USA) with 10% FBS (Thermo Fisher Scientific, Waltham, MA, USA). To obtain undifferentiated HaCaT cells the medium was exchanged for DMEM without calcium (Life Technologies—Thermo Fisher Scientific, Waltham, MA, USA) supplemented with calcium-free 10% FBS as described in [21], and the cells were cultured in these conditions for at least 14 days. Primary human keratinocytes, NHEK (PromoCell, Heidelberg, Germany), were cultured in Keratinocyte Growth Medium 2 (PromoCell, Heidelberg, Germany) with growth supplement as described in [16]. To induce differentiation of NHEKs and of undifferentiated HaCaT cells the respective culture media were supplemented with 1.8 mM CaCl_2_ (final concentration) and the cells were cultured for 72 h.

### 2.2. SUV39H1 Knockout Cells

HaCaT cells were transfected, using Lipofectamine3000 (Invitrogen - Thermo Fisher Scientific, Waltham, MA, USA), with pCMV-Cas9-GFP plasmids (Sigma Aldrich, St. Louis, MO, USA) encoding caspase Cas9, GFP, and one of the four gRNA sequences (gRNA1-4): gRNA1-CGTGTGTTGCAAGTCTTCTTGG, gRNA2-TTCCTCTTAGAGATACCGAGGG, gRNA3-GTTCCTCTTAGAGATACCGAGG, or gRNA4-GATCTTCTTGTAATCGCACAGG, targeting the second exon of *SUV39H1*. Twenty-four hours post-transfection, the cells were sorted on a BD FACSaria II flow cytometer (BD Biosciences, San Jose, CA, USA) equipped with the BD FACS Diva software v6.1.2. using a 488 nm blue laser, and single-cell colonies were established in 96-well plates. The expanded cell clones were examined by dot blot and those that appeared negative for the SUV39H1 protein expression were further analyzed by Western blot. To assess the presence of mutation(s) in *SUV39H1* in these clones, nuclear DNA was isolated according to a standard procedure and amplified in a PCR reaction with the following primers: F: GGGGTTCAAAGCACATTTCTG and R: TGTGTTTTCAGGGTCAAAGGA encompassing the second exon of *SUV39H1*. The PCR product was sequenced by Genomed S.A. (Warsaw, Poland).

### 2.3. Sample Preparation, Western Blot, and Dot Blot

Whole-cell lysates were prepared as described in [21]. A histone-containing fraction was obtained by acidic extraction following the protocol available at (https://www.abcam.com/protocols/histone-extraction-protocol-for-western-blot). Protein concentration was determined using a protein assay (Bio-Rad, Hercules, CA, USA). Samples containing 80 µg protein were precipitated first by adding a 1:10 volume of 100% trichloroacetic acid for 30 min on ice and, after a 10 min centrifugation at 12,000× *g*, cold acetone for 16 h at −20 °C. Samples were subjected to SDS-PAGE and immunoblotting. Blots were examined with antibodies recognizing SUV39H1 (rabbit polyclonal, Thermo Fisher Scientific, 1:1000), histone H3 (rabbit polyclonal, 1:1000, Merck Millipore, Burlington, MA, USA), H3K9me3 (rabbit polyclonal, 1:1000, Thermo Fisher Scientific, Waltham, MA, USA), and β-actin (HRP-conjugated mouse monoclonal, Sigma Aldrich, 1:20,000). In the case of dot blots, cells (1 × 10^5^) from individual cell clones were collected and lysed in 10 µl of lysis buffer (Promega—Cell Signaling technology, Danvers, MA, USA) for 30 min on ice with occasional vortexing, and the resulting lysates were directly transferred on nitrocellulose and processed as in a Western blot.

### 2.4. Immunocytochemistry (ICCH)

ICCH was performed as described in [22] using a rabbit anti-H3K9me3 antibody (Merck Millipore, 1:500) followed by a secondary anti-rabbit IgG antibody coupled with Alexa Fluor 488 (Invitrogen—Thermo Fisher Scientific, Waltham, MA, USA). Cells were observed under a confocal spinning disc microscope (Zeiss, Jena, Germany) using 63× oil objective and visualized by ImageJ.

### 2.5. Quantitative Reverse Transcription PCR (RT-qPCR)

Total RNA was isolated using the Extractme kit (Blirt DNA, Gdansk, Poland). One microgram of RNA was reverse transcribed using M-MLV reverse transcriptase (Sigma Aldrich, St. Louis, MO, USA). mRNA levels were then analyzed by RT-qPCR using the SYBRGreen system with 18S rRNA as a standard. The primers used are listed in Supplementary Table S1. The results were analyzed by absolute quantification with a relative standard curve and normalized to 18S rRNA using the comparative ΔΔCt method.

### 2.6. Chromatin Immunoprecipitation (ChIP)

ChIP was performed using the Imprint Chromatin Immunoprecipitation Kit (Sigma Aldrich, St. Louis, MO, USA) and a Bioruptor Plus apparatus (Diagenode, Denville, NJ, USA). HaCaT cells were treated with 1% formaldehyde for 20 min and the crosslinking reaction was then stopped with the addition of glycine at 125 µM final concentration. Cells were collected in PBS, centrifuged at 400× *g* for 5 min, and the subsequent steps were performed according to the kit manufacturer’s instructions using 25 × 10^5^ cells per sample. All antibodies used in the assay were rabbit polyclonal raised against the following antigens: acetylated histone H3 (acH3) (Merck Millipore, Burlington, MA, USA), histone H3, histone H3 trimethylated on lysine 27 (H3K27me3), and histone H4 trimethylated on lysine 20 (H4K20me3) (all three from ThermoFisher Scientific, USA). Four micrograms of each antibody were added, i.e., immobilized in the well, per sample. The same amount of rabbit IgG fraction was added to control samples. A PCR reaction was conducted using the following forward/reverse primer pairs: LCE1A—5′-TGTGAAAGCATCTGACAAACAA-3′/5′-TGTTCAGGAGCTGAAGGAGA-3′, LCE1B—5′-TCCCAGCCAGTGTAGAGGATA-3′/5′-CTGCAAAGGAAGTTGGAGGAAA-3′, and LCE1E—5′-TTCAGGGTGTGAAGACATATT-3′/5′-GCAGGACATCTCGGCAGTAG-3′. The PCR products were resolved on agarose gels and quantified by densitometry. The intensity of the IgG band was subtracted from all other values (bands of lower intensity was were not included in the analysis). Finally, the intensity of bands corresponding to the examined histone modifications was normalized to the intensity of the H3 band from the same gel.

### 2.7. Cell Adhesion Assays

To measure cell adhesion to the dish surface, WT and SUV-KO HaCaT cells were counted using an EVE cell counter (NanoEntek, Seoul, South Korea) and seeded at the density of 2 × 10^5^ per well of a 24-well plate. After 2 h, the wells were washed with PBS to remove cells that did not adhere and a fresh medium containing 0.5 mg/mL of 3-(4,5-dimethythiazol-2-yl)-2,5-diphenyl tetrazolium bromide (MTT) was added. Cells were cultured for 2 h, washed with PBS, and absorption was measured in 100 µl of DMSO at 570 nm in an Infinite 200 PRO plate reader (Tecan, Mannedorf, Switzerland). The cell-to-cell adhesion assay was carried out essentially as described by [23] except that MTT was used as a staining dye and cells were counted as above prior to seeding. Briefly, WT and SUV-KO HaCaT cells were seeded at a density of 5 × 10^4^ cells per well of a 96-well plate and cultured overnight. MTT (0.5 mg/mL) was then added for 2 h to stain the cells. After that the cells were trypsinized and seeded again on a monolayer of confluent WT HaCaT cells, and cultured for 1–2 days before the experiment in wells of a 96-well plate. Absorbance was measured after 2 h of cell culture and then again, in a fresh portion of medium, after non-adherent cells were removed by PBS. The absorbance ratio represented the ratio of adherent versus the total amount of MTT-stained cells seeded on a monolayer of WT HaCaT cells.

### 2.8. Statistical Analysis

Statistical data analysis was performed using a two-tailed Student’s *t*-test. Experiments were performed at least 3 times with 3 technical replicates. A *p*-value ≤0.05 was set as a threshold for statistical significance. Figure graphs were prepared using GraphPad software. Statistically significant differences between samples are indicated by horizontal bars and either asterisks or *p*-values.

## 3. Results

### 3.1. Generation of HaCaT Cells with Knockout of SUV39H1 Histone Methyltransferase Using the CRISPR/Cas9 Approach

**Figure 1:**
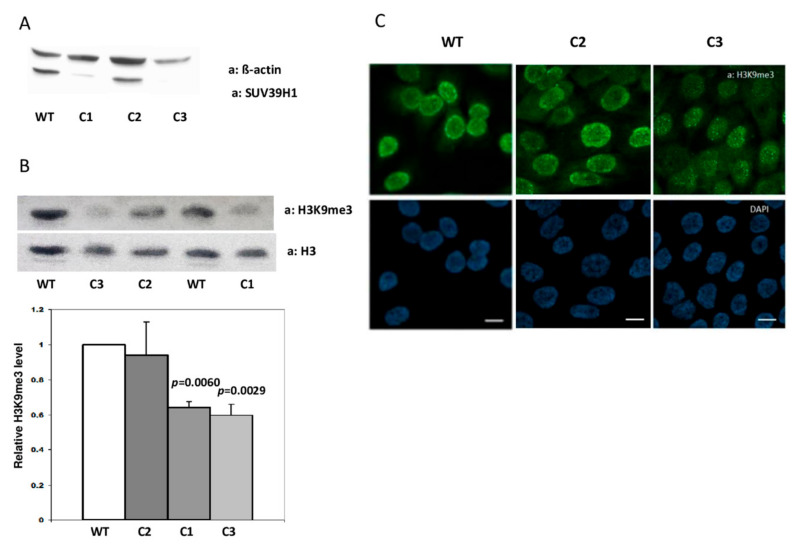
Characteristics of SUV39H1-KO HaCaT cells. (**A**) Representative Western blot showing expression of SUV39H1 in three single-cell clones (C1–C3) derived from HaCaT cells transfected with pCMV-Cas9-GFP plasmids. (**B**) Upper panel—representative Western blot showing the level of histone H3K9me3 in C1, C3 (SUV-39H1-KO mutation), C2 (no mutation), and WT HaCaT cells. Lower panel—statistical analysis of the results of n = 3 Western blots; bars represent mean ± SEM. (**C**) Representative confocal microscopy images of WT HaCaT cells and clones C2 (no mutation) and C3 (SUV39H1-KO mutation) stained for H3K9me3 (green). Nuclei are stained with DAPI (blue). Scale bar: 10 µm.

To obtain HaCaT cells devoid of SUV39H1 activity, we transfected them with pCMV-Cas9-GFP plasmids, each encoding caspase Cas9, GFP, and one of the four gRNA sequences (gRNA1-4) listed in Materials and Methods. Single cells, sorted 24 h post-transfection based on GFP fluorescence, were allowed to expand and protein lysates were then preliminarily examined by the dot-blot method. Cell clones that appeared negative for SUV39H1 expression on dot blots (not shown) were further propagated and examined by Western blot. An example of this analysis is shown in Figure 1A. The analyzed clones, two apparently negative (C1, C3) and one positive (C2) for SUV39H1 expression were further examined by sequencing of the PCR-amplified genomic DNA fragment comprising the second exon of *SUV39H1*. The sequencing results documented a wild-type sequence for clone C2 and the deletion of one nucleotide (T) in clones C1 and C3, which led to a frame shift and premature stop codon (Supplementary Figure S1).

To check the specificity and physiological consequences of the single nucleotide deletion in the second exon of *SUV39H1*, clone C2 (no mutation in *SUV39H1*) and clones C1 and C3 (with deletion) were examined for the content of histone H3 modified by trimethylation on lysine 9 (H3K9me3) using a Western blot. This analysis demonstrated no difference in H3K9me3 content (evaluated as H3K9me3/total H3 ratio) between WT HaCaT cells and clone C2 and about 40% lower H3K9me3 content in clones C1 and C3 bearing the deletion (Figure 1B). Clones C2 and C3 were further compared to H3K9me3 content and localization by means of immunofluorescence staining. Microscopic images confirmed the results of the Western blot in that they showed a similar intensity and staining pattern of H3K9me3 in C2 and WT HaCaT cells when compared to the overall weaker staining of H3K9me3 in the nuclei of cells from the C3 clone, which was especially evident at the nuclear boundary (Figure 1C). Thus, both analyses demonstrated that the observed decrease in H3K9me3 level is an inherent feature of HaCaT cell clones bearing a *SUV39H1* knockout mutation (clones C1 and C3) and not merely an off-target effect introduced due to transfection with pCMV-Cas9-GFP plasmids (clone C2).

### 3.2. Changes in Expression of Selected EDC Genes and Non-EDC-Encoded Keratinocyte Differentiation Markers in SUV39H1-KO HaCaT Cells

**Figure 2:**
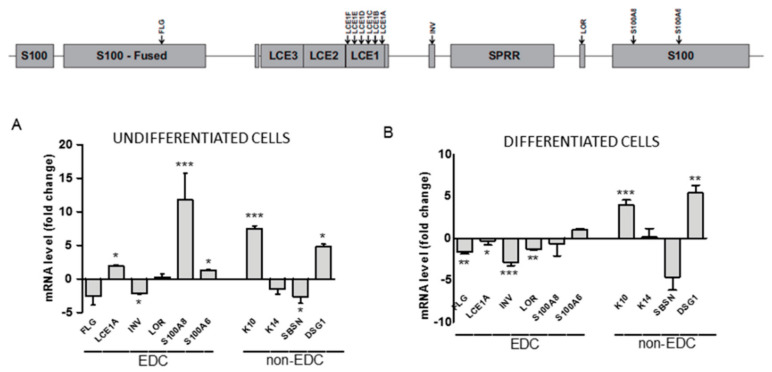
Changes in mRNA level of selected EDC and non-EDC genes in SUV39H1-KO HaCaT cells. Upper panel—schematic representation of EDC; position of the examined genes is indicated by arrows. Lower panel—fold difference in gene expression in (**A**) undifferentiated and (**B**) differentiated SUV39H1-KO HaCaT cells over WT HaCaT cells. The bars represent mean ± SEM calculated from n = 3–5 (depending on the gene) RT-qPCR experiments. Each experiment consisting of three technical replicates and was performed on a different batch of RNA. Statistically significant differences are indicated by asterisks; * *p* ≤ 0.05, ** *p* ≤ 0.01, *** *p* ≤ 0.001.

Epigenetic modifications of chromatin regulate many cellular and organismal processes, amongst them cell differentiation. To examine the impact of impaired H3K9 trimethylation on keratinocyte differentiation, we compared the relative expression of selected genes known to be activated or downregulated throughout the process in undifferentiated and differentiated WT and SUV39H1-KO (clone C3) HaCaT cells. The analysis included genes located in the EDC (Figure 2, upper panel): *FLG*, *LCE1A*, *INV*, *LOR*, *S100A8*, *S100A6* (encoding filaggrin, late cornified envelope protein 1A, involucrin, loricrin, and the S100A8 and S100A6 proteins, respectively) and non-EDC genes: *SBSN*, *K14*, *K10*, *DSG1* (encoding suprabasin, keratin 14, keratin 10, and desmoglein 1, respectively). Among these genes, expression of *K14*, *S100A6*, and *SBSN* is restricted to the basal and suprabasal epidermal layer so the corresponding proteins can be considered markers of undifferentiated keratinocytes or keratinocytes at an early stage of differentiation [5,24,25], whereas LCE1A, INV, LOR, S100A8, DSG1, and FLG are long-established markers of differentiated cells. RT-qPCR analysis demonstrated a differential response of particular genes to a lower level of H3K9 methylation.

As presented in Figure 2, the expression of some genes (*S100A6*, *K14*, *LOR*) appeared to be not or only slightly affected by the impairment in H3K9 trimethylation both in undifferentiated and differentiated cells. *FLG*, *INV*, and *SBSN* expression exhibited a slight differentiation state-independent downregulation. On the other hand, the mRNA level of four proteins, namely, K10, DSG1, LCE1A, and S100A8, all encoding markers of differentiated keratinocytes, was clearly upregulated. Interestingly, while *K10* and *DSG1* expression was upregulated in both undifferentiated and differentiated cells, upregulation of *LCE1A* and *S100A8* was observed only in undifferentiated SUV39H1-KO HaCaT cells. This finding indicates that H3K9me3 could suppress these genes by binding to their promoters in undifferentiated but not in differentiated cells. To check whether this was really the case, we concentrated on LCE1 genes, i.e., a subgroup of LCE genes, since such an analysis could provide additional information on a possible co-regulation of highly homologous adjacent genes.

### 3.3. Changes in Histone Modifications at LCE1 Promoters during Keratinocyte Differentiation

**Figure 3:**
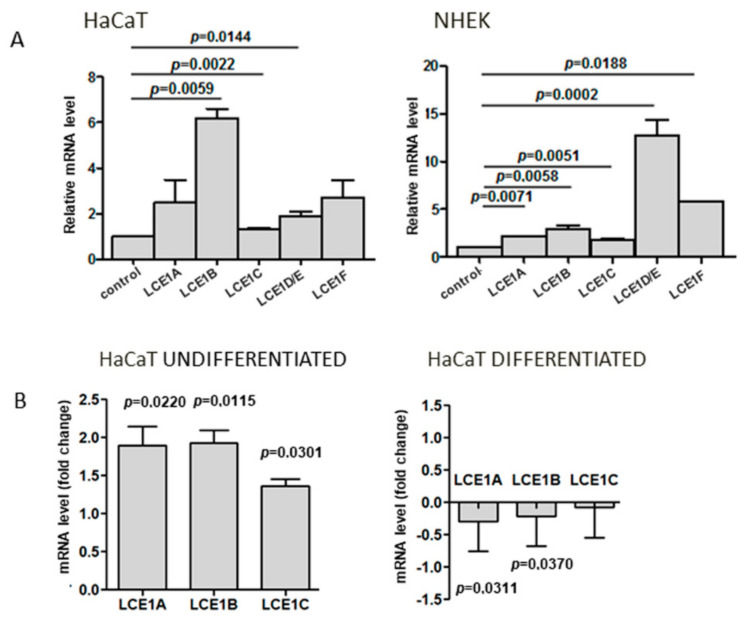
Expression of *LCE1* genes. (**A**) Upregulation of *LCE1A-F* transcripts in differentiated HaCaT cells (left panel) and primary human keratinocytes, NHEK (right panel), in comparison to undifferentiated cells (control). (**B**) Fold difference in expression of *LCE1A*, *LCE1B*, and *LCE1C* in undifferentiated (left panel) and differentiated (right panel) SUV39H1-KO HaCaT cells over WT HaCaT cells. The bars represent mean ± SEM calculated from the results of n = 3–5 (depending on the gene) RT-qPCR experiments. Each experiment consisted of three technical replicates and was performed on a different batch of RNA. Statistically significant differences are indicated by horizontal bars and the respective *p*-values.

Since there are only limited data concerning the expression of human LCE genes as a function of keratinocyte differentiation, we first assessed the relative level of mRNA of genes forming the LCE1 gene subgroup within EDC. The analysis was performed for both primary human keratinocytes and HaCaT cells. The level of all studied transcripts, that is, LCE1A, LCE1B, LCE1C, LCE1D/E (impossible to discern due to high sequence similarity), and LCE1F, increased upon differentiation of both cell types, and the increase was statistically significant in the majority of cases (*p* ≤ 0.05) (Figure 3A).

To see whether the expression of other *LCE1* genes was altered in a similar way as that of *LCE1A* in SUV39H1-KO cells, we quantified the *LCE1B* and *LCE1C* transcripts that were the most and the least upregulated during HaCaT cell differentiation by RT-qPCR. As shown in Figure 3B, as with *LCE1A*, these genes exhibited higher expression in undifferentiated SUV39H1-KO cells than in their WT counterparts. On the other hand, practically no difference in the expression of these genes was observed between differentiated WT and SUV39H1-KO HaCaT cells.

We then verified by means of chromatin immunoprecipitation (ChIP) whether the increase in *LCE1* expression was accompanied by changes in the pattern of epigenetic modifications of histone H3 present on the promoters of *LCE1* genes. We analyzed the relative level of H3K9me3 bound to *LCE1A*, *LCE1B*, and *LCE1E* promoter regions in undifferentiated and differentiated HaCaT keratinocytes. Furthermore, using appropriate antibodies, we extended the ChIP analysis to include acetylated histone 3 (acH3), histone 3 trimethylated on lysine 27 (H3K27me3), and histone 4 trimethylated on lysine 20 (H4K20me3).

**Figure 4:**
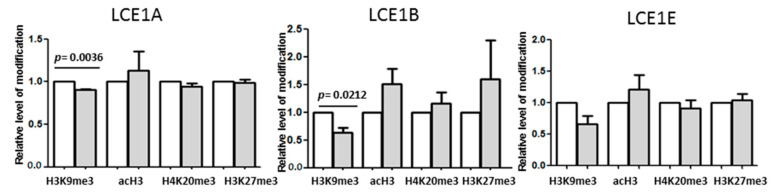
ChIP analysis of histone modifications present on promoters of selected *LCE1* genes. The graphs represent relative level of histone H3 or H4 modifications (H3K9me3, H3ac, H3K27me3, H4K20me3) on promoters of *LCE1A*, *LCE1B*, and *LCE1E* genes in undifferentiated (white bars) and differentiated (gray bars) HaCaT cells. The bars represent mean ± SEM calculated from n = 3–5 (depending on the modified histone) experiments. Statistically significant differences are indicated by horizontal bars and the respective *p*-values.

As shown in Figure 4, the pattern of histone modifications did not change much upon differentiation. In particular, despite a clear rising tendency, the level of H3 acetylation, an activating modification, did not increase significantly. There was, however, a consistent drop in the level of H3K9me3 on all three gene promoters studied that proved to be statistically significant in the case of *LCE1A* and *LCE1B*. Thus, the results of the ChIP analysis complied with the difference in the *LCE1* expression pattern between WT and SUV39H1-KO HaCaT cells shown in Figure 3B in that they demonstrated a higher prevalence of H3K9me3 on *LCE1* promoters in undifferentiated than in differentiated HaCaT cells. Together, these results indicate that during keratinocyte differentiation, the expression of *LCE1A*, *LCE1B*, and *LCE1E* genes is jointly and directly regulated by H3K9 methylation.

### 3.4. Changes in Cell-to-Cell and Cell-to-Surface Adhesion of SUV39H1-KO HaCaT Cells

Beside differences in gene expression, the SUV39H1-KO HaCaT cells exhibited some subtle morphological features that differentiated them from their WT counterparts. Namely, SUV39H1-KO HaCaT cell colonies seemed more compact, with clearly defined edges, and the cells were less susceptible to trypsin treatment, which often produced cell sheets rather than single cells. This observation concurred with the increased level of DSG1 mRNA in both undifferentiated and differentiated SUV39H1-KO cells (Figure 2). DSG1 is the main component of desmosomes, that is, specialized structures involved in the tight packing of keratinocytes in the upper epidermal layers [26]. This prompted us to investigate the efficiency of cell-to-cell adhesion in the studied cells. To do so, we performed an adhesion assay essentially as described in [23], in which we compared the adhesion of WT HaCaT cells and SUV39H1-KO HaCaT cells to a monolayer of WT HaCaT cells.

**Figure 5:**
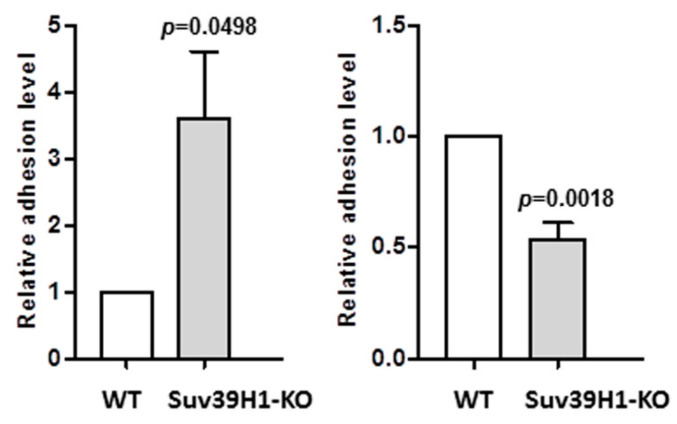
Adhesion assay. Left panel—adhesion of WT (white bar) and SUV39H1-KO (gray bar) HaCaT cells to a confluent monolayer of WT HaCaT cells. Right panel—adhesion of WT (white bar) and SUV39H1-KO (gray bar) HaCaT cells to the dish surface. The MTT absorbance measured in WT HaCaT cells (control) containing wells was taken as 1. The bars represent mean ± SEM calculated from the results of n = 3 experiments, each consisting of three technical replicates.

As shown in Figure 5 (left panel), SUV39H1-KO HaCaT cells adhered more efficiently to WT HaCaT cells than did the WT cells. On the contrary, adhesion to the dish surface, which is mediated by hemidesmosomes, i.e., integrin-containing structures typical for undifferentiated basal keratinocytes [27], was more efficient in the case of WT HaCaT cells (Figure 5, right panel). The decrease in surface adhesion coincided with a lower expression of integrin β1 in SUV-KO HaCaT cells (Supplementary Figure S2). Altogether, changes in adhesion properties support the notion that SUV39H1-KO HaCaT cells possess characteristics of more differentiated keratinocytes than WT HaCaT cells.

## 4. Discussion

Trimethylation of histone H3 on lysine 9 is the primary modification involved in constitutive heterochromatin organization and stability [28]. Accordingly, any manipulation of the expression level of SUV39H1 or SUV39H2, two methyltransferases that catalyze H3K9 trimethylation, ultimately results in alterations in the structure and stability of satellite repeats and other chromatin regions containing repetitive elements [28,29] and in changes in telomere length [30,31]. The presence of H3K9me3 is not, however, limited to heterochromatin. In embryonic stem cells, H3K9me3 was found to co-localize with H3K4me3 and H3K27me3 in euchromatin regions, mostly at promoters of developmentally expressed genes [32]. In differentiated human cells, H3K9me3-rich chromatin regions were more numerous and extended than in embryonic stem cells, and they spanned a higher number of gene promoters, especially promoters of developmental genes [33] and S-phase genes involved in cell proliferation [34]. Such transcriptional restriction of a defined set of genes limits pluripotency and determines cell fate upon differentiation [33].

Epidermal differentiation entails a gene expression program that, through orchestrating multiple biochemical and morphological changes in keratinocytes, ensures the formation of a tight epidermal barrier. This process can be seriously dysregulated in such skin-related processes as wound healing, skin aging, and various skin pathologies. Even though the involvement of epigenetic components has been identified in many of the above phenomena [35,36], the regulation of epidermal differentiation by epigenetic factors, including histone modifications, remains relatively unexplored [13]. Quiescent epidermal stem cells in the interfollicular epidermis are characterized by a high global level of inhibitory modifications, H3K9me3, and H4K20me3, and low levels of H4K20me1 and acetylated histone H4 [37]. An increase in histone H4 hyperacetylation and H4K20 mono- and then di-methylation (H4K20me2) was observed upon Myc-induced transient cell amplification. As regards gene promoters, changes in the level of H3K4me3 and H3K27ac, markers of active promoters, were found between keratinocyte progenitors and differentiated cells [18]. The inhibitory histone modification, H3K27me3, is present on promoters of late-expressed genes in embryonic epidermal progenitors [19] while in differentiated keratinocytes on promoters of genes actively transcribed in progenitor cells [18]. As for H3K9me3, no changes in total H3K9me3 level and only limited redistribution of this histone mark were observed during keratinocyte differentiation [38]. The level of SUV39H1 and EZH2 histone methyltransferases was found to diminish along with keratinocyte differentiation [19,38]. These findings are in agreement with the observation that most epidermal gene promoters are active in all stages of keratinocyte differentiation [18], which implies that probably only relatively small or local changes in epigenetic modifications may trigger large changes in gene expression.

In this study we showed that the knockout of SUV39H1 histone methyltransferase resulted in a substantial reduction in the level of H3K9me3, the apparent reorganization of H3K9me3-dense chromatin regions, and changes in the expression of several keratinocyte-specific genes. Of note, the expression of genes associated with undifferentiated keratinocytes, that is, K14, S100A6, and SBSN, was either unaltered or even slightly diminished in SUV39H1-KO HaCaT cells, whereas several genes (*LCE1*, *S100A8*, *K10*, and *DSG1*) encoding keratinocyte differentiation markers were upregulated. Enhancement in the expression of only some of the studied differentiation-associated genes points to a defined topology of the H3K9me3 mark imposed by SUV39H1. Interestingly, changes in the expression of epidermal genes were also noted in the nasal epidermis of dogs with loss-of-function mutation in *SUV39H2* [39,40]. Namely, *INV* and *LOR* expression was slightly diminished and that of *K10* increased, similar to SUV39H1-KO HaCaT cells, but the expression of DSG1 was not affected. The latter observation suggests that, apart from common sites, these two enzymes may modify histone H3 at different genomic locations. Of note, double (*SUV39H1/SUV39H2*) knockout mice appeared to have no obvious skin phenotype [28]. Thus, it seems that changes in the level of epidermal gene expression elicited by the loss of H3K9 methyltransferases do not compromise the function of the epidermis, possibly due to various compensatory mechanisms [41].

Among genes with upregulated expression in SUV39H1-KO HaCaT cells, we could discern those whose expression changed dependent on (*LCE1* genes, *S100A8*) or independent of (*K10*, *DSG1*) the differentiation stage. We assumed that genes upregulated exclusively in undifferentiated HaCaT cells could be direct targets of H3K9me3. To check this hypothesis, we concentrated on the highly homologous and functionally related genes of the LCE1 subcluster. LCE1 genes were shown to be regulated by the same transcription factors [41,42] and responded to the same environmental stressor [43], so it was highly probable that they also shared the same mechanism of epigenetic regulation. Indeed, ChIP analysis showed direct binding of H3K9me3 to promoters of *LCE1A*, *LCE1B*, and *LCE1E* genes in undifferentiated keratinocytes and partial release of this binding, or demethylation, in differentiated cells. Joint regulation of *LCE1* by H3K9 methylation is reminiscent of other gene clusters and may reflect the propensity of H3K9me3 to bind to repetitive sequences [30]. Likely, the same direct regulatory mechanism, i.e., the presence of H3K9me3 on the gene promoter, is responsible for the regulation of *S100A8*. S100A8, together with its dimerization partner, S100A9, belongs to the class of damage-associated molecular pattern molecules (DAMPs) that are expressed and released from numerous cell types upon various stresses [44]. S100A8 mRNA increases upon the differentiation of primary human keratinocytes [15], but the overall protein level in the suprabasal layer of a normal epidermis is low. It can, however, increase many-fold following wounding, UV exposure, etc., and is also elevated in psoriasis and other skin diseases [5,44]. Our result suggests that H3K9me3 demethylation or release from an *S100A8* promoter may contribute to this acute increase in the level of S100A8.

The expression of two of the studied genes, *K10* and *DSG1*, was also upregulated by SUV39H1 knockout but independent of the stage of keratinocyte differentiation. We believe that this could be due to the overall chromatin rearrangement following substantial H3K9me3 loss. Still, enhancement in the expression of these genes while other studied genes remained unaffected points to a defined topology of the H3K9me3 mark imposed by SUV39H1. Of note, in the nasal epidermis of dogs with loss-of-function mutation in SUV39H2, *INV* and *LOR* expression was slightly diminished and that of *K10* increased, similar to SUV39H1-KO HaCaT cells, but interestingly, the expression of DSG1 was not affected [43,44]. The latter observation suggests that, apart from common sites, these two enzymes may modify histone H3 at different genomic locations. DSG1 is implicated in numerous skin diseases [26], therefore an insight into how its expression is regulated may have a prospective clinical value.

## 5. Conclusions

SUV39H1 knockout in HaCaT keratinocytes caused a marked decrease in the level of H3K9me3 and entailed selective rather than global changes in the expression of differentiation-associated genes. Some genes appeared to be directly regulated by H3K9 trimethylation due to the binding of H3K9me3 to their promoters, whereas changes in the expression of other genes probably resulted from the overall alterations in chromatin evoked by H3K9me3 depletion. On the whole, the rise in expression following SUV39H1 knockout concerned mainly genes of the middle/late stage of differentiation and reinforced the differentiation process in comparison to wild-type HaCaT cells. A more differentiated phenotype of HaCaT cells with SUV39H1 knockout was also evidenced by their enhanced cell-to-cell adhesion, which correlated with the upregulated expression of desmoglein 1, a component of desmosomes. Thus, local loss of the H3K9me3 mark imposed by SUV39H1 may represent one of the epigenetic changes accompanying epidermal differentiation.

## Supplementary Materials

The following are available online at https://www.mdpi.com/2073-4409/9/12/2628/s1. Figure S1. Upper panel: chromatograms showing sequencing results for C2 (WT) and C1/C3 (mutated) cell clones. The mutated fragment is indicated by a black horizontal bar. Lower panel: the corresponding nucleotide and amino acid sequences. Supplementary Figure S2: relative level of integrin β1 mRNA in WT (white bar) and SUV-KO (gray bar) HaCaT cells. * *p* ≤ 0.05. Supplementary Table S1: the list of primers used in RT-qPCR. The primers for LCE1 genes are as in [43].
