# Supplementary material for: Effect of SUV39H1 Histone Methyltransferase Knockout on Expression of Differentiation-Associated Genes in HaCaT Keratinocytes
Source: Cells. 2020 Dec 7;9(12):2628. doi: 10.3390/cells9122628 (PMC7762351; doi:10.3390/cells9122628)
Supplement: Supplementary file 1 [file cells-09-02628-s001.pdf]

Suppl. Fig. S1

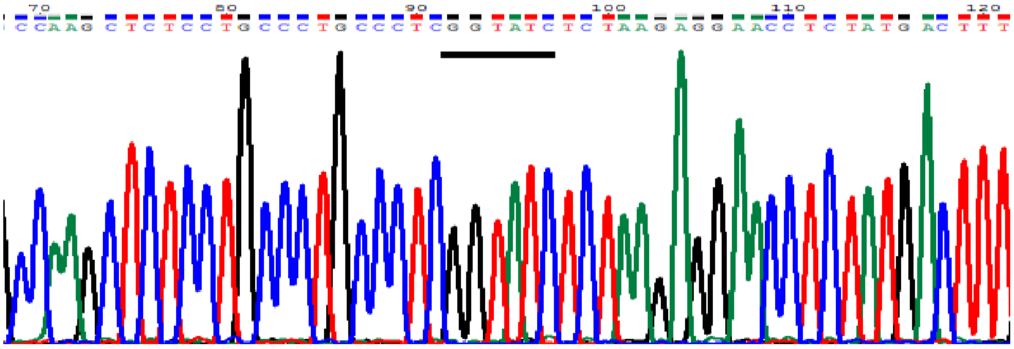

WT sequence

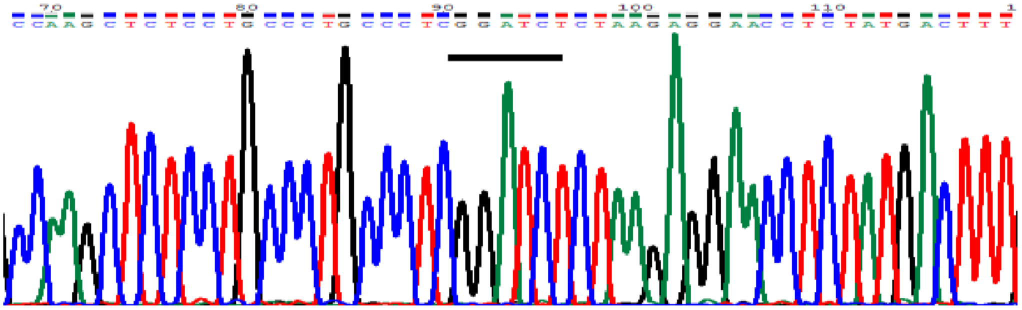

Mutated sequence

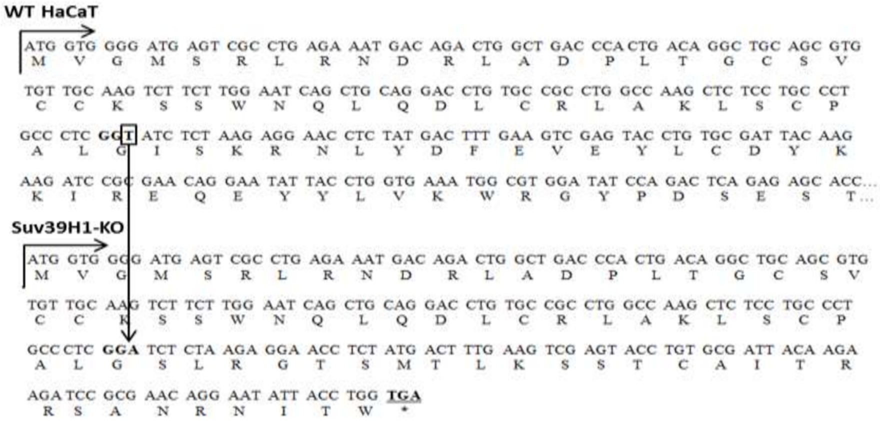

Suppl. Fig. S2

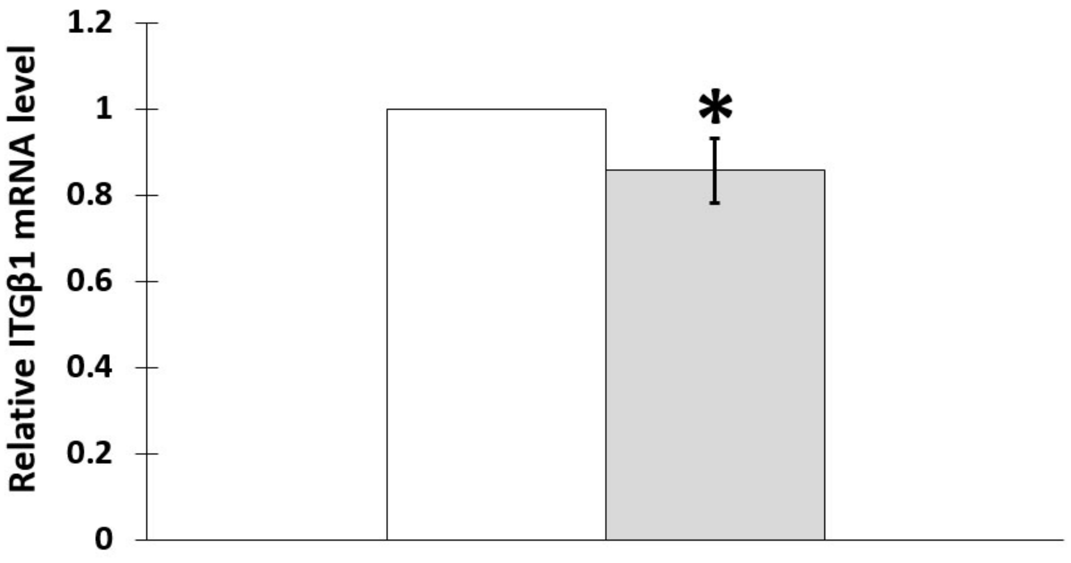

Suppl. Table S1

Gene	Forward primer	Reverse primer
**LCE1A**	5’- TGCAAGAGTGGCTGAGATGC -3’	5’-AGACAACACAGTTGGTGTCAGG-3’
**LCE1B**	5’-TGAGCCTAGAAGAACACAA-3’	5’-GAAGGAGAAGGAAGACGG-3’
**LCE1C**	5’-GAATCCAGGACCGCAAACTG-3’	5’-TGGACCTGTGAGCCTCTCAG-3’
**LCE1D/E**	5’-TGAATAGCTGAGAGGTTCCAGC-3’	5’-CAGCCATGGATCTGCAGAAG-3’
**LCE1F**	5’-CCACACCGAAGTGCC-3’	5’-GCTGCAGCAGGAAGAG-3’
**FLG**	5’-CACAAGATTCTGCGTATCACTCAGG-3’	5’-GCCTTTCAGTGCCCTCAGATTG-3’
**LOR**	5’-GGAGTTGGAGGTGTTTTCCA-3’	5’-CAAACCTCGGGTAGCATCAT-3’
**K10**	5’-TGGCCTTGAAACAATCCCTG-3’	5’-AGCGACCTTCTGTTTCTGCC-3’
**K14**	5’-GAGTGTGGAGCCGACATCAA-3’	5’-GCCTCTCAGGGCATTCATCTC-3’
**INV**	5’-GGGTGGTTATTTATGTTTGGGTGG-3’	5’-GCCAGGTCCAAGACATTCAAC-3’
**DSG1**	5’-TCCCATAGTTGATCGAGAGGTCAC-3’	5’-CTGCGTCAGTAGCATTGAGTATC-3’
**S100A6**	5’-ATGGCATGCCCCCTGGATCA-3’	5’-CAGAGCTTCATTGTAGATCAA-3’
**S100A8**	5’-CCGAGTGTCCTCAGTATATCAGGAA-3’	5’-ACGCCCATCTTTATCACCAGAAT-3’
**SBSN**	5’-TCTGCTCTGCGTCCTGATAG-3’	5’- GAAATGAAAGGCACCCAAAA-3’
**ITGβ**	5’-GAAGGGTTGCCCTCCAGA-3’	5”-GCTTGAGCTTCTCTGCTGTT-3’
**18S rRNA**	5’-CTCAACACGGGAAACCTCAC-3’	5’-CGCTCCACCAACTAAGAACG-3’
